# The Multidimensionality of Welfare State Attitudes: A European Cross-National Study

**DOI:** 10.1007/s11205-012-0099-4

**Published:** 2012-06-12

**Authors:** Femke Roosma, John Gelissen, Wim van Oorschot

**Affiliations:** 1 Department of Sociology, Tilburg University, P.O. Box 90153, 5000 LE Tilburg, The Netherlands; 2 Department Methodology and Statistics, Tilburg University, P.O. Box 90153, 5000 LE Tilburg, The Netherlands

**Keywords:** Welfare state, Welfare attitudes, Welfare legitimacy, Public opinion, Cross-national research, European social survey

## Abstract

When evaluating the various aspects of the welfare state, people assess some aspects more positively than others. Following a multidimensional approach, this study systematically argues for a framework composed of seven dimensions of the welfare state, which are subject to the opinions of the public. Using confirmatory factor analyses, this conceptual framework of multidimensional welfare attitudes was tested on cross-national data from 22 countries participating in the 2008 European Social Survey. According to our empirical analysis, attitudes towards the welfare state are multidimensional; in general, people are very positive about the welfare state’s goals and range, while simultaneously being critical of its efficiency, effectiveness and policy outcomes. We found that these dimensions relate to each other differently in different countries. Eastern/Southern Europeans combine a positive attitude towards the goals and role of government with a more critical attitude towards the welfare state’s efficiency and policy outcomes. In contrast, Western/Northern Europeans’ attitudes towards the various welfare state dimensions are based partly on a fundamentally positive or negative stance towards the welfare state.

When evaluating the various aspects of the welfare state, people assess some aspects more positively than others. For example, people often support substantial state involvement while simultaneously being critical of the welfare state’s level of bureaucracy and perceived lack of efficiency (Svallfors 2010). Attitudes towards a complex phenomenon such as the welfare state are likely to be ambivalent or even contradictory (Svallfors 1991); therefore, several scholars have suggested that the welfare state should be assessed as a multidimensional phenomenon and that welfare attitudes should be measured accordingly (Svallfors 1991; Sihvo and Uusitalo 1995; Gelissen 2000; Van Oorschot and Meuleman 2011).

However, there is limited research on welfare attitudes from a multidimensional perspective, and only a few single-country studies have simultaneously examined attitudes towards the multitude of welfare state dimensions (Van Oorschot and Meuleman 2011; Gelissen 2000; Sihvo and Uusitalo 1995; Svallfors 1991). Most of these studies question whether attitudes towards the welfare state result from distinct attitude patterns regarding the various welfare state dimensions or result from one underlying attitude towards the welfare state. These studies come to different conclusions. In general, the studies agree that attitudes towards the welfare state are indeed multidimensional, but the studies are inconclusive about the structure of the attitude patterns. These inconclusive results can have at least three different causes. First, they can be the result of differences between countries. A particular country can have a greater range or a different set of welfare state attitudes and attitude patterns than another country, and this difference warrants a comparative analysis of the multidimensionality of welfare state attitudes. Second, these differences may be due to varying operational definitions of the welfare state dimensions used in the studies. In fact, the choice of dimensions to analyse is mostly data-driven, given that most studies lack the theoretical arguments for selecting welfare state dimensions. The existing studies give only limited reasons for the salience of particular welfare state dimensions. Lastly, different conclusions may be the result of using particular methods. For example, Svallfors (1991) used an Exploratory Factor Analysis (EFA) with the assumption of orthogonal factors to find five underlying attitude patterns in the data, and Sihvo and Uusitalo (1995) performed an EFA on separate groups of items to validate their theoretical dimensions and subsequently correlate these dimensions. Van Oorschot and Meuleman (2011) have argued that these two studies do not really test the multidimensionality of welfare attitudes, because in their choice of methods, they isolate the items that estimate the latent construct. As a result, the shared variance between the items is not taken into account. Therefore, van Oorschot and Meuleman argue for an approach that uses a Confirmatory Factor Analysis (CFA) as a methodological tool for examining all items in one empirical model (e.g., Gelissen 2000; Sabbagh and Vanhuysse 2006).

This paper further explores the multidimensional approach in welfare attitude research by contributing to the literature in two ways. First, because of the lack of theoretical arguments for discerning relevant welfare state dimensions, the aim of this study is to select the various dimensions of the welfare state by theoretical reasoning and relate them in a systematic manner in one coherent framework. Second, the study seeks to investigate the empirical tenability of the proposed conceptual framework using new comparative data on welfare state attitudes from the European Social Survey (2008) for 22 European countries. The large number of countries allows for a more stringent test of the proposed dimensionality of welfare state attitudes and its validity across countries than in existing single country studies. To examine these attitude structures we follow Van Oorschot and Meuleman’s (2011) recommendation to use a Confirmatory Factor Analysis to study the multidimensionality of welfare state attitudes. Thus, our research questions are as follows: (1) “What dimensions of the welfare state can theoretically be distinguished?” (2) “What is the level of European public support for these dimensions?” (3) “Is this public support for the welfare dimensions based on a unidimensional attitude or on multidimensional attitudes?” (4) “What are the cross-national differences in public support and attitude structures among European countries?”

## Dimensions of the Welfare State

### The Welfare State and its Legitimacy

What welfare state dimensions can we distinguish? To answer this question, let us start with the proposition that the welfare state is the institutionalised answer to the distributional justice question, “How (should) a society or group (…) allocate its scarce resources or product to individuals with competing needs and claims?” (Roemer 1996).

In the literature, this central question of distributional justice is believed to follow from two historical developments: (1) Kant’s idea that people are equal and have an equal right to earthly goods (Fleischacker 2004), which led to democratisation and the overthrow of the old class system (Roller 1995) and (2) industrialisation, which led to dependence on the market for survival and created the need for social security (Roller 1995; Esping-Andersen 1990). These developments made redistribution a matter of justice and an urgent societal problem (Fleischacker 2004). The welfare state’s main goals are to address these developments by promoting social justice to mitigate unjust inequalities (Spicker 2000; Fleischacker 2004) and by providing protection against the market’s rigidity through a social security system (Esping-Andersen 1990). To achieve these goals, the welfare state redistributes resources and becomes the institutional embodiment of regulated redistribution. This redistribution focuses on not only redistributing means and goods, but also reallocating life chances by giving people equal opportunities and a certain socio-economic status. In other words, the welfare state regulates individuals’ life chances by redistributing income, risks and services (Mau 2003).

The welfare state’s redistribution process must be embedded in a shared idea of social justice and fairness to be legitimate. Because the welfare state answers the question of distributional justice, the welfare state itself should be a legitimate solution that is based on a shared idea of justice and fairness. Rothstein (1998) identifies three conditions for welfare state legitimacy. First, the public should believe that the goals and substance of the policy programs are just and fair and that politicians need to justify their policy decisions under those terms. This condition, which Rothstein calls *substantive justice*, justifies what the state *should* do. Second, the redistribution process must meet a *just distribution of burden*. The public may support the general goals of welfare programs, but they must believe that their fellow citizens will also contribute to these programs and that the burdens of this contribution will be distributed fairly. This condition determines what contributions to the welfare state *should* be shared. The third condition is the existence of *procedural justice*. People must believe that the implementation of programs follows their goals and is effective and efficient. Implementation should be simple, cheap and directed towards making cheating difficult. It justifies what the state *can* do (or is doing), instead of what it *should* do (Rothstein 1998). Using these three conditions for welfare state legitimacy, Rothstein combines the questions ‘what ought to be’ and ‘what can be’ into one analysis of the welfare state design and thus reveals the underlying logic of welfare state legitimacy: if the welfare state meets public expectations about what the state *should* do. If the welfare state *can* be implemented fairly, then it will be regarded as legitimate and will generate its own support (Rothstein 1998).

### Dimensions of the Welfare State

We use the definition of the welfare state as a redistributor of life chances, along with Rothstein’s conditions of legitimacy, as the backbone of our conceptual framework of various welfare state dimensions. This framework follows the policy process logic: from formulating policy goals, through policy implementation, to policy outcomes.

**Fig. 1:**
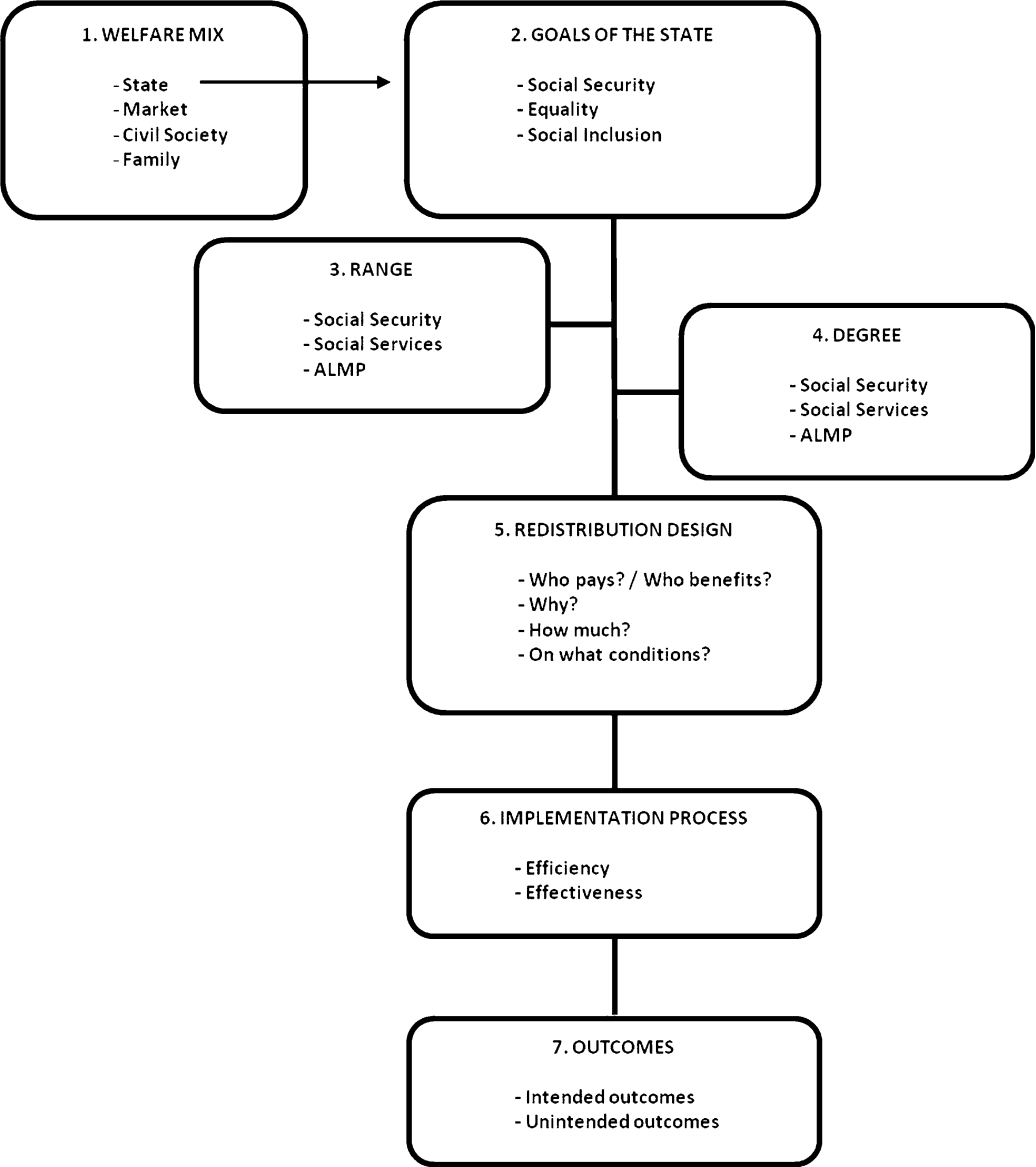
The dimensions of the welfare state

We identify seven welfare state dimensions that overlap dimensions established in previous studies on the multidimensionality of the welfare state (Van Oorschot and Meuleman 2011; Sihvo and Uusitalo 1995; Svallfors 1991); however, these dimensions are defined more precisely in relation to our theoretical starting points. A model of these seven dimensions is presented in Fig. 1.

Before describing the dimensions of the welfare state itself, we distinguish the *welfare mix* dimension, which recognises that, in addition to the state, there are other redistributive institutions whose roles, relative to those of the state and to each other, are important matters of debate. Should the state redistribute, or should we leave this up to the family, the market or private institutions, such as the church (Barr 1993)? We will not include this dimension of the debate in our study, since our interest here is in the redistributional characteristics of the welfare state.

The second dimension focuses on the main redistributional goals of the state. This *goals* dimension refers to the overarching goal of the welfare state and relates to the two welfare state goals that developed through democratisation and industrialisation: The goal to impose some kind of social justice in which all people are considered of equal worth (Roller 1995; Fleischacker 2004), thus promoting either a liberal idea of equality of opportunity or a more egalitarian idea of equality of outcomes (Esping-Andersen 1990), and the goals of social security and protection of the public against the rigidity of the market (Esping-Andersen 1990; Roller 1995). In the last few decades, new normative frameworks have been developed for the role of the welfare state in which welfare policies emphasise activation of people for the labour market or other forms of societal participation. Based on this “welfare to workfare” trend, the welfare state is sometimes relabelled the *enabling state*, which has the underlying goal of including people in society through participation rather than allowing them to become completely dependent on social provisions (Gilbert 2004). This goal makes the redistribution of job opportunities important. In summary, we can define three prominent welfare state goals: providing social security, imposing equality (of opportunity and/or outcome) and promoting social inclusion through participation.

Next, we distinguish a *range* dimension and a *degree* dimension, labels that were introduced by Roller (1995). Given that the welfare state uses redistribution to achieve its goals, these dimensions reflect the areas of life and society in which the state should redistribute (range), and how much it should redistribute (degree). These are usually the core dimensions of welfare attitudes research, since most opinion surveys contain questions specifically related to these dimensions. Together with the *goals* dimension, they form the substance of the welfare state and relate to Rothstein’s condition of substantive justice and what the state *should* do. The *range* dimension refers to the areas of life in which the state should redistribute (Roller 1995). Research practices in defining the range of government responsibilities differ and are mostly data driven (see, for example, Cnaan 1989; Roller 1995; Andress and Heien 2001; Blekesaune and Quadagno 2003). Here, we distinguish three *range* subdimensions: social benefits, social services and active labour market policies. The government can be responsible for various social benefits, such as old age pensions, unemployment benefits, sick leave, social assistance and various other social services including health care, education, and child care services (Muuri 2010). To achieve the goal of participation, individual social services and active labour market policies (ALMP) have been implemented (Gilbert 2004). The *degree* dimension refers to how much effort the government should expend redistributing in certain policy areas or “the intensity of government activity within a policy area” (Roller 1995). This dimension is often operationalised in terms of preferences for the size of welfare spending in particular social policy areas (Pettersen 1995; Papadakis and Bean 1993; Cnaan 1989; Sihvo and Uusitalo 1995). The *degree* dimension can apply to the same three subdimensions indicated for *range*: social benefits, social services and active labour market policies.

The next dimension regards the actual design of the redistribution process and relates to issues such as “Who should benefit from the redistribution in different policy areas”, “Who should contribute to it, and for what reasons and on what conditions?”, “Who should carry the burdens of redistribution?”, “What groups are deserving of what types of benefits and on what conditions?” (Gilbert and Terrell 2010; Van Oorschot 2006). This *redistribution dimension* relates to Rothstein’s legitimacy condition of a just distribution of burdens and to what the welfare state *should* do (Rothstein 1998).

The *implementation* dimension relates to Rothstein’s condition of procedural justice, i.e., implementation in a fair manner. This dimension refers to what the welfare state *can* do or is actually doing and has two subdimensions: *efficiency* and *effectiveness*. *Efficiency* considers questions such as “Are administrations and services not spilling money, delivering on time, and easy to understand?” (Rothstein 1998), “Are they accountable and accessible?” (Gilbert and Terrell 2010). *Effectiveness* pertains to whether benefits and services reach the legitimate beneficiaries with limited abuse and non-take up of benefits (Halvorsen 2002; Ervasti 1998; Edlund 1999; Svallfors 1991).

Finally, we distinguish an *outcomes* dimension. Although this dimension is not reflected in Rothstein’s conditions of legitimacy, we believe that there are relevant attitudes about the performance of the welfare state and that these attitudes contribute to its legitimacy: If the welfare state performs according to expectations and desires, its legitimacy will be greater. The *outcome* dimension is divided in two sub-dimensions: *intended outcomes* and *unintended outcomes* (Roller 1995). On the one hand, intended outcomes relate to the welfare state’s goals: Are equality, social security and labour activation attained? Is inequality reduced and social security provided? On the other hand, intended outcomes relate to outcomes of the redistribution process: Are benefits generous enough, are services satisfactory? Unintended outcomes refer to economic and moral consequences of the welfare state (Van Oorschot 2010). The former relates to the financial burden that the welfare state places on the government budget and its consequences for tax levels and the economy, and the latter relates to possible moral hazards. People can rely on the welfare state too much by shunning their own responsibility or becoming lazy or individualistic (Van Oorschot 2010).

## Welfare Attitudes: Unidimensional or Multidimensional?

Our multidimensional perspective on the welfare state assumes that people have different, and possibly contradicting, attitudes towards the various dimensions of the welfare state. The competing view, assuming unidimensionality, holds that people draw upon one general attitude towards the welfare state as a whole, and deduce their particular opinions on specific welfare related issues from that: they are either pro- or anti-welfare state, and this is reflected in each separate opinion. Existing empirical research on the issue tends to find support for the multidimensionality of welfare state attitudes (Svallfors 1991; Sihvo and Uusitalo 1995; Van Oorschot and Meuleman 2011), but these findings are limited as they are based on national studies. In the remainder of this study, we empirically investigate whether the unidimensionality or the multi-dimensionality hypothesis finds support when tested on recent large-scale cross-national data on welfare attitudes.

## Data and Methods

### Data

To answer our empirical research questions, we analysed data from the European Social Survey 2008 wave (ESS, Round 4). This wave contains a module on welfare attitudes that is currently the most extensive cross-national dataset for measuring welfare attitudes available. Therefore, these data can be considered a unique opportunity, allowing us to measure most—but unfortunately not all—dimensions of our conceptual framework. We selected 26 items by which we measured five welfare state dimensions (excluding the *welfare mix* and *redistribution* dimensions), divided into ten subdimensions. Table 1 gives a summary of the selected dimensions and their operational definitions.^1^

**Table 1:** Operationalisation of welfare state dimensions—ESS data 2008 Round 4

Dimension	Scale		Number of items	ESS code
Goals	1–5	(Strongly) agree to reduce income levels	1	B30
Range	0–10	Government should be responsible for…	6	D15–D20
Degree	0–10	Increase taxes and social spending	1	D34
Efficiency	0–10	Social systems are (extremely) efficient	2	D30–D31
Effectiveness/abuse	1–5	Disagree that people abuse benefits/services	1	D42
Effectiveness/underuse	1–5	Disagree that people underuse benefits/services	1	D41
Outcomes goals	1–5	(Strongly) agree that goals are reached	3	D22, D23 D26
Outcomes policy	0–10	Benefits/services are (extremely) good	6	B28, B29, D11–D14
Outcomes economic	1–5	(Strongly) disagree WS harms economy	2	D21, D25
Outcomes moral	1–5	(Strongly) disagree WS is bad for morals	3	D27–D29

We selected 22 European countries (N = 41.507): Belgium (BE), Bulgaria (BG), Switzerland (CH), Cyprus (CY), Czech Republic (CZ), Germany (DE), Denmark (DK), Estonia (EE), Spain (ES), Finland (FI), France (FR), United Kingdom (GB), Croatia (HR), Hungary (HU), Latvia (LV), Netherlands (NL), Norway (NO), Poland (PL), Portugal (PT), Sweden (SE), Slovenia (SI), Slovakia (SK).^2^

The ESS contains no items that allow measuring attitudes on aspects of the *welfare mix*. The data include one item that measures the *goals* dimension, i.e., support for reducing income levels (related to the goal of equality). For the *range* dimension, six items were selected, regarding the extent to which the government is responsible for ensuring jobs, health care, a reasonable standard of living for the old and for the unemployed, child care and for providing paid care leave. The *degree* dimension was measured with one item asking about respondents’ support for either “increasing taxes and spending more on social benefits and services” or “decreasing taxes and spending less”. We did not include items for the *redistribution* dimension since the available items were measured at the nominal measurement level and therefore were not suitable for further analysis in structural equation modelling with continuous variables.

The *implementation* dimension was operationalised with its two sub-dimensions: *efficiency* and *effectiveness*. *Efficiency* contains two items regarding how efficient the health care system and tax system (in handling queries on time, avoiding mistakes and preventing fraud) are perceived. *Effectiveness* was measured by people’s perception of *abuse* (“many people manage to obtain benefits to which they are not entitled”) and *underuse* (“many people get fewer benefits than they are legally entitled to”) of welfare benefits. Because abuse and underuse did not form a reliable scale (average Cronbach’s alpha for 22 countries was only 0.32), we included both these items separately.

The outcomes of the welfare state were measured by four subdimensions: *outcomes*-*goals*, *outcomes*-*policy*, *outcomes*-*economic*, and *outcomes*-*moral*. People’s opinions about whether welfare state goals are met were measured with three items: Do social benefits and services lead to a more equal society, less poverty and make it easier to combine work and family life? *Outcomes*-*policy* asked whether the policy outcomes are satisfactory with six items: What do you think of the state of education, the state of health care, the standard of living of the old, of the unemployed, the provisions of affordable child care services, and opportunities for young people to find a job? *Outcomes*-*economic* measured the unintended consequences for the economy with two items: Does the welfare state place too great a strain on the economy, and costs businesses too much in taxes and charges? Finally, *outcomes*-*moral* measured whether people believe the welfare state has unintended moral consequences with three items: Does the welfare state makes people lazy, less willing to care for one another and less willing to look after themselves and their family? All variables are coded such that a higher score represents a more pro-welfare attitude.

### Methods

The analysis proceeded in five steps. First, we addressed the issue of cross-national measurement invariance of attitudes towards dimensions of the welfare state using multi-group Confirmatory Factor Analysis (CFA) (Byrne 1998). Next, we analysed public support for the different (sub)dimensions, thus indicating the extent of public support for the dimensions of the welfare state. In the third step, we inspected how strongly the dimensions are correlated, which subsequently led us to a formal test of the one- and multidimensionality hypothesis of welfare state support. We followed the same approach as Gelissen (2000), Sabbagh and Vanhuysse (2006) and Van Oorschot and Meuleman (2011) by using CFA as a methodological tool to model underlying attitude structures. In the fourth step, we examined the shared variation of all dimensions, and finally, we examined the differences between the European countries. We compared their mean scores on the dimensions and analysed differences in their attitude patterns.

## Results

### Measurement Invariance

With multi-group CFA, we tested whether the attitudes towards welfare state dimensions, which are assumed to be latent constructs, are measurement invariant across countries. Invariance would indicate the cross-national comparability of these constructs. For CFA, at least three items per latent construct are needed (Steenkamp and Baumgartner 1998). This requirement means that for several dimensions we cannot formally assess measurement invariance across countries. Nonetheless, we additionally present the findings for these dimensions because we would like to provide the reader with as complete a picture as possible of welfare attitudes using the best cross-national data currently available. We note, however, that the information regarding country comparisons on these dimensions, given the current impossibility of performing a strict test of their measurement invariance, should be considered more carefully. For the dimensions that have sufficient items (*range* and *intended* and *unintended outcomes*), measurement invariance is assessed.

At least partial scalar invariance is required to compare the means of latent variables (Steenkamp and Baumgartner 1998; Davidov 2008). For the *range* dimension partial scalar invariance holds, with a moderate fit statistic for the RMSEA and good fit statistics for the CFI.^3^ For the *outcomes* dimensions, we tested one structural model with four (sub)dimensions (*outcomes*-*goals*, *outcomes*-*policy*, *outcomes*-*economic* and *outcomes*-*moral*) and their indicators to be measurement invariant across countries. This model indicated partial scalar invariance.^4^ Sum scores were calculated for each scale.^5^

### Public Support for the Different Dimensions of the Welfare State

In Table 2, we present the percentages of people that score above and below the scale midpoint for the pooled dataset of 22 European countries.

**Table 2:** European support for welfare state dimensions

Dimension	% pro-welfare attitudes^a^	% anti-welfare attitudes^b^
Goals	71	14
Range	94	4
Degree	35	29
Efficiency	54	34
Effectiveness/abuse	17	62
Effectiveness/underuse	21	52
Outcomes goals	63	25
Outcomes policy Output	42	53
Outcomes economic	34	42
Outcomes moral	45	42

^a^Pro welfare: % >3/>5 (depending on the scale, 1–5 and 0–10, respectively; see Table 1)

^b^Anti welfare: % <3/<5 (depending on the scale, 1–5 and 0–10, respectively; see Table 1)

Table 2 shows that support for the *goals* and *range* dimensions is very high: Most Europeans believe that the government should redistribute more to reduce income differences and be responsible for various social security benefits and socials services. This result is fully in line with previous research (Blekesaune and Quadagno 2003; Roller 1995; Gelissen 2000; Andress and Heien 2001; Papadakis and Bean 1993). Concerning the *degree* dimension people are more reserved: 29 % prefer lower taxes and lower social spending, but 35 % want the government to raise taxes to spend more on social benefits and services. Note that 36 % of Europeans choose the scale midpoint: They believe that the degree of government spending is sufficient. In studies in which survey questions about government spending do not mention the related consequence of increasing taxes, higher levels of support for government spending are usually found (Gelissen 2000; Pettersen 1995; Cnaan 1989; Papadakis and Bean 1993). Turning to the *implementation* dimension, we see that about half of respondents believe that health care and tax authorities are efficient, and one-third does not. The European public is most critical about the welfare state’s effectiveness: About 50–60 % perceive substantial abuse and underuse of welfare benefits. This result has also been found in other studies (Ervasti 1998; Halvorsen 2002; Svallfors 1991; Edlund 1999). Perceptions of cheating may undermine support for the welfare state in general and form a risk for its legitimacy because of a lack of procedural justice.

Compared to welfare state goals and design, people are on the whole less positive about its outcomes. The majority of respondents in these European countries do believe that the welfare state attains its main goals in preventing poverty and promoting equality (*outcomes goals*), but more than half believe that policy outcomes such as benefit levels and the quality of services are insufficient. About 40 % consider the welfare state to harm the economy and cause moral hazards, while about the same proportion disagrees. These *outcomes* dimensions, of which people are more critical than *range* and *degree* issues, have thus far not been systematically analysed in the literature (Van Oorschot et al. 2012), with a generally too-positive picture of welfare state support in the literature as a result. A large proportion of respondents are particularly unsatisfied with the policy outcomes of the welfare state: Benefits are deemed insufficient and services inadequate. Such disappointment with the welfare state’s outcomes may lead to decreasing legitimacy, but the empirical evidence indicates there is still support for a large role of the government. Moreover, comparing the percentages of pro- and anti-welfare attitudes on all subdimensions, the European public is overall more positive than negative about the welfare state.

### One- or Multidimensional Welfare Attitudes?

To obtain a first impression of whether welfare state support is unidimensional or rather multidimensional and to see how the different dimensions relate to each other, we inspected the correlations between the dimensions. Moreover, we used this correlation matrix to adjust the structural latent factor model developed next. Table 3 shows the correlations between the sum-scores of the dimensions.

**Table 3:** Correlations between dimensions (sum scores)

	Goals	Range	Degree	Effic.	Abuse	Under- use	Outc. goals	Outc. policy	Outc. eco.	Outc. mor.
Goals	1.000									
Range	0.288	1.000								
Degree	0.085	0.144	1.000							
Efficiency	−0.057	−0.022	0.171	1.000						
Effective/abuse	−0.074	−0.031	0.149	0.161	1.000					
Effective/underuse	−0.212	−0.177	−0.014	0.126	0.215	1.000				
Outcomes goals	0.008	0.001	0.157	0.247	0.108	0.036	1.000			
Outcomes policy	−0.205	−0.162	0.122	0.538	0.171	0.238	0.276	1.000		
Outcomes economic	0.071	0.157	0.274	0.033	0.234	0.001	−0.004	0.022	1.000	
Outcomes moral	0.062	0.196	0.223	0.058	0.286	0.001	0.009	−0.007	0.437	1.000

The correlations are low, which indeed suggests that welfare state attitudes are multidimensional and that scale-scores do not result from one general welfare state attitude. Nevertheless, some dimensions are related. Not surprisingly, there is a correlation between the *unintended outcomes* dimensions: the coefficient for the correlation between *outcomes*-*economic* and *outcomes*-*moral* is 0.437. People who believe that the welfare state has negative economic consequences also believe that the welfare state has negative moral effects. Since these dimensions are theoretically related, we included a specific (second-order) latent factor representing *unintended outcomes* in our proposed structural model. Furthermore, Table 3 also shows a relatively high correlation of 0.538 between the dimensions *efficiency* and *outcomes*-*policy.* Because this result could theoretically be expected—an inefficient system will cause poor policy outcomes—we allowed a correlation between these dimensions in the structural model. Finally, we imposed a correlation in the structural model between *unintended outcomes* and the expected level of abuse. Abuse of social benefits is a form of ineffective redistribution—an implementation problem—but abuse can also be considered to result from moral hazards and thus to be an unintended consequence of the welfare state. We note that later inspection of modification indices also suggested that inclusion of this correlation is warranted.

The moderate correlation of 0.288 between the *goals* and the *range* dimensions may result from the fact that both dimensions refer to the substance of the welfare state and to Rothstein’s concept of substantive justice. The negative correlations between both these dimensions and the *outcomes*-*policy* dimension are interesting: people who are positive towards a greater role of the welfare state are also more critical of its outcomes. We will examine these relations more thoroughly later. We do not make any further adjustments to our proposed structural model, except for allowing some correlations between individual items that are for different substantive reasons related. For instance, the item that measures people’s perceptions about the role of government in providing health care and the item that measures people’s opinion on the state of the health care system are supposed to share some variance related to their subject (see Appendix Table 8).

**Fig. 2:**
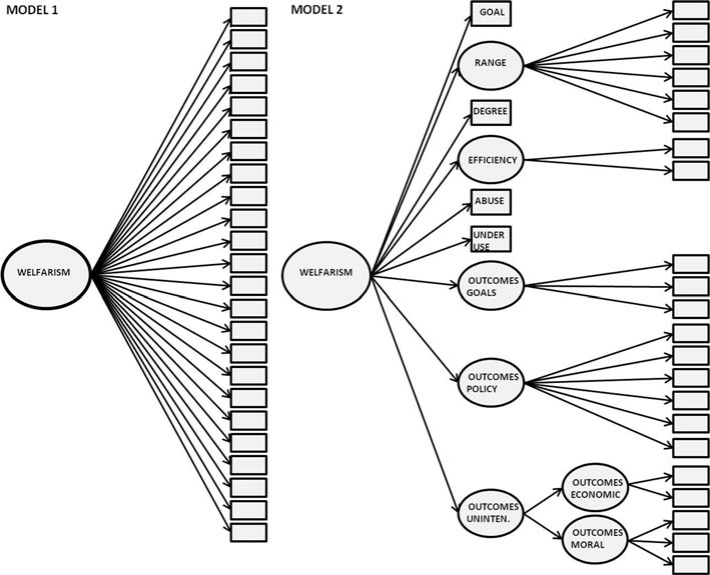
Hypothesised structural models

We tested the unidimensionality or multidimensionality hypothesis by specifying two CFA models with competing attitude structures (See Fig. 2). Model 1 hypothesised that the 26 selected items load on one latent factor that represents a general attitude towards the welfare state, which we call *welfarism*. Model 2 included six latent variables for the dimensions that are measured by more than one item (*range*, *efficiency*, *outcomes goals*, *outcomes policy*, *outcomes economic* and *outcomes moral*) and four single-item measurements (for *goals*, *degree*, *effectiveness*-*abuse* and *effectiveness*-*underuse*). As explained earlier, the model further assumed a second-order latent factor for the *unintended outcomes*. We also included a third-order factor to inspect the relationship between the dimensions. The third order factor defines what the latent dimensions have in common. Its factor loadings present the strength and direction (positive or negative) of the relationship between the latent dimensions and the third-order factor. Because all the (latent) variables that pertain to a particular domain of the welfare state also all relate to the notion of welfare, we expected that the domain-specific factors should have some variance in common, which will be detected by the third-order factor. However, if the multidimensionality hypothesis holds, this general third-order factor should have only a weak impact (i.e., weak to moderate loadings) on the domain-specific factors. Moreover, we were interested in the ways in which dimensions in European countries are related to each other. Including a third order factor was a parsimonious way to inspect these relationships in a structural latent factor model, instead of allowing correlations between all the dimensions. Van Oorschot and Meuleman (2011) chose this approach for the Dutch data and defined *welfarism* as a pro- or anti-welfare state attitude that may explain the shared variation between the latent dimensions. We also call this factor *welfarism*, but we note that it may be interpreted differently in different countries depending on how the dimensions are interrelated.

In Table 4, we present the results for the pooled sample of European countries. We see that Model 1 has a poor fit, whereas Model 2 has a good fit (RMSEA of <0.05 and a CFI of >0.9).^6^ An assessment of measurement invariance indicated that configural measurement invariance (i.e., equal factor structures) (Steenkamp and Baumgartner 1998; Davidov 2008) holds for Model 2, but not for Model 1: In all countries, Model 2 shows a good or acceptable model fit, whereas Model 1 has a poor fit in all countries.^7^

**Table 4:** Goodness of fit statistics for the latent factor models (Pooled sample N = 41.507)

	Model 1	Model 2
Chi-squared	199,065.591	23,184.322
Df	299	279
*P* value	0.000	0.000
RMSEA	0.127	0.044
CFI	0.350	0.925
BIC	3,787,205.578	3,611,536.981

### The Third-Order Factor Welfarism

The first order factor loadings (of the individual items on the latent dimensions) and second-order factor loadings (of *outcomes economic* and *outcomes moral* on *unintended outcomes*) of Model 2 are presented in Table 8 in the Appendix, and show an expected pattern with loadings >0.40. The third-order factor loadings of the latent dimensions on *welfarism* are presented in Table 5. How should we interpret this factor for the pooled sample of European countries? We see that the dimensions *goals*, *range*, *underuse*, and *outcomes*-*policy* load high on *welfarism*, while *degree*, *abuse*, *outcomes goals* and *unintended* *outcomes* have a substantially weaker relationship with *welfarism*.

**Table 5:** Third order factor loadings Model 2 (standardised)

	Welfarism
Goals	0.498
Range	0.577
Degree	0.077
Efficiency	−0.321
Effectiveness/abuse	−0.201
Effectiveness/underuse	−0.427
Outcomes goals	−0.214
Outcomes policy	−0.603
Outcomes unintended	0.250

The dimensions that refer to the substance of the welfare state (the *should* dimensions) are positively correlated to this factor, while the dimensions that evaluate the implementation process and the welfare state’s intended outcomes have a negative relationship. Therefore, we argue that in terms of European welfare attitudes, *welfarism* represents a general idea that the welfare state should do more, since it does not perform well enough. This rather general attitude weakly influences specific welfare opinions via the domain-specific latent dimensions, which are empirically more clearly discernible.

### Cross-National Differences

After empirically inspecting the dimensionality of welfare attitudes of the European public, we proceeded to examine European cross-national differences by showing how public attitudes and the relationships between the latent dimensions differ between European countries.

Table 6 presents the order of the countries based on their country means for attitudes towards the dimensions. We estimated the latent means for the six latent welfare-dimension variables, while for the four single-item dimensions, we report their observed means. Standard deviations show that especially the *range*, *efficiency* and *outcomes policy* dimensions vary greatly between countries. On these dimensions, we particularly see differences between Western and Northern European welfare states (white areas), on the one hand, and Eastern and Southern European welfare states (grey areas), on the other.^8^ However, this distinction also appears on the *goals*, *underuse* and *outcomes goals* dimensions. In general, the Eastern and Southern European countries more strongly endorse a greater role of the government, but at the same time, they are more critical of their welfare states’ efficiency and effectiveness (especially underuse of benefits) and their intended outcomes (*outcomes goals* and *outcomes policy*). The Czech people seem to be an exception because they are less in favour of a greater role of the government (*goals* and *range*), do not believe that there is much underuse, and are less critical towards policy outcomes. This exceptional position of the Czech Republic on attitudes towards the role of government was also found in other welfare attitude research (Lipsmeyer 2003).

**Table 6:**
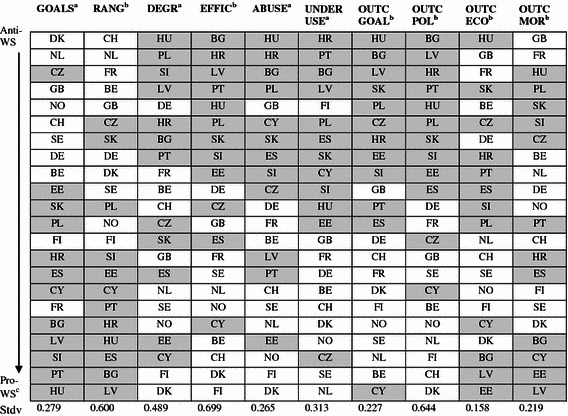
Order of European countries’ scores on (latent) dimensions

*Grey areas* Countries of Eastern/Southern Europe. *White areas* Countries of Western/Northern Europe

^a^Sum scores based on pooled sample

^b^Latent means based on the structural model (model 2)

^c^See Table 1 for definition of pro-welfare state and anti-welfare state

To scrutinise these country differences more closely and assess the relationship between the latent dimensions, we estimated a structural model for both regions separately. We labeled Finland, Sweden, Norway, Denmark, the Netherlands, Germany, Belgium, France, Switzerland and United Kingdom as Western/Northern European welfare states (N = 19.717) and Bulgaria, Croatia, Czech Republic, Cyprus, Estonia, Hungary, Latvia, Poland, Portugal, Slovakia, Slovenia and Spain as Eastern/Southern European welfare states (N = 28.424). We again determined the model fit of Model 1 and 2, but now for both subsamples separately. To compare structural models for both regions, we first assessed measurement invariance. For identification purposes, we excluded the latent factor for *unintended* *outcomes* and imposed a correlation between *outcomes economic* and *outcomes moral*. Under this condition, we find scalar level measurement invariance,^9^ which allows us to compare both the factor loadings and the means of the latent constructs.

Table 7 shows that in both regions, Model 2 has a good RMSEA and a relatively good CFI (for Western/Northern countries, it is slightly under the cut-off point of >0.9 for the second multidimensional model). Model 1 is rejected for both Western/Northern and Eastern/Southern countries. For both regions, we find a multidimensional attitude structure.

**Table 7:** Goodness of Fit statistics for the latent factor models

	Western/Northern Europe		Eastern/Southern Europe	
	Model 1	Model 2	Model 1	Model 2
Chi-squared	74,754.443	13,119.293	108,328.051	10,594.762
Df	299	279	299	279
*P* value	0.000	0.000	0.000	0.000
RMSEA	0.112	0.048	0.129	0.041
CFI	0.347	0.887	0.357	0.939
BIC	1,775,689.422	1,714,252.055	1,966,540.354	1,869,006.849

Considering the differences in country scores on the subdimensions, we expect the relationships between the dimensions to differ for the country groupings. How do these relations differ? The answer to this question is revealed by the factor loadings of *welfarism* and latent means of the dimensions that are shown in Table 8 in the Appendix, which shows substantial differences between both country groupings. In the Western/Northern countries, *welfarism* has a positive relationship towards all latent dimensions (except for a weak negative relation with *underuse*). *Welfarism* here represents either a positive or negative attitude towards the welfare state in general, which influences single welfare attitudes indirectly via latent dimensions. Van Oorschot and Meuleman (2011) found a similar underlying construct. There is an especially strong relationship with the *range*, *degree* and *unintended* *outcomes* dimensions. Respondents’ ideas about the role of government, the level of welfare spending and possible negative outcomes of the welfare state are influenced by their general feeling towards the welfare state, whereas their ideas about the policy outcomes are less influenced by a general welfare attitude.

**Table 8:** Standardised factor loadings—Structural model (Model 2) (Pooled sample N = 41.507)

	Goals	Range	Degree	Efficiency	Abuse	Underuse	Outcomes goals	Oucomes policy	Outcomes unintended	Outcomes economic	Outcomes moral
Goals	x										
Range—jobs		0.664									
Range—health		0.637									
Range—old		0.697									
Range—unemployed		0.671									
Range—child care		0.667									
Range—care leave		0.635									
Degree			x								
Efficiency—health care				0.698							
Efficiency—taxes				0.555							
Abuse					x						
Underuse						x					
Outc goals—poverty							0.675				
Outc goals—equality							0.776				
Outc goals—work/fam							0.587				
Outc policy—educ.								0.524			
Outc policy—health								0.589			
Outc policy—old								0.618			
Outc policy—unempl.								0.581			
Outc policy—childcare								0.468			
Outc policy—yng jobs								0.590			
Outcomes economic									0.750		
Outcomes moral									0.812		
Outc eco—economy										0.685	
Outc eco—businesses										0.661	
Outc moral—lazy											0.817
Outc moral—no care											0.740
Outc moral—look after											0.717
Third and second order loadings on welfarism	0.498	0.577	0.077	−0.321	−0.201	−0.427	−0.214	−0.603	0.250	x	x

Goodness of Fit statistics: Chi Squared 23 184.322, Df 279, RMSEA 0.044 and CFI 0.925

*Correlations between items:* range health—range old/range child care—range care leave/range old—outc. pol health/range unempl—outc. pol unempl/outc. goals work/fam—outc. mor look after/outc. pol educ—outc. pol health/outc. pol old—outc. pol unempl/outc.pol unempl—outc. pol yng jobs/effic. health—outc. pol health/outc.mor lazy—outc. eco economy/abuse—underuse/abuse—outc.mor. lazy

*Correlations with latent factors:* outc. unintended—abuse/efficiency—outc.pol

In the Eastern/Southern European countries, *welfarism* seems to be a construct that should be interpreted differently and that can be compared in some ways to the results for the pooled sample. Here *welfarism* shows a substantial negative relationship with the *efficiency, effectiveness* and *outcomes policy* dimensions and a substantial positive relationship with the *goals* and *range* dimensions. For Eastern/Southern European welfare attitudes, *welfarism* embodies support for a larger role of the welfare state (*should* dimensions) but also a critical attitude towards its efficiency, effectiveness and policy outcomes (*is* dimensions). The idea that the welfare state performs poorly and that its role should (therefore) be increased influences people’s opinions on single welfare attitude questions. A comparison of the latent means confirms the differences with the Western/Northern countries: Respondents in Eastern/Southern European countries support a larger role of the government but are also more critical towards the welfare states’ efficiency, their benefit level and quality of social services (i.e., policy outcomes).

## Conclusion and Discussion

In this paper, we contributed to previous research on multidimensionality of the welfare state by systematically arguing for a conceptual framework of seven dimensions. Moreover, we tested this framework on cross-national data on welfare attitudes from the European Social Survey.

We conclude, first, that if we want to understand the social legitimacy of the welfare state in all its aspects, we should examine welfare state attitudes from a multidimensional perspective. According to our empirical analysis, welfare state legitimacy cannot be adequately investigated and understood by only looking at people’s general idea about the welfare state or by examining attitudes from a unidimensional perspective. Attitudes towards some welfare state dimensions clearly differ from attitudes to other dimensions. A second conclusion is that, generally, people in European countries are very positive about the welfare state’s goals and range, while simultaneously feeling critical about its efficiency, effectiveness and policy outcomes. Perceived ineffectiveness of the welfare state and the perception of abuse and underuse of welfare state benefits and services are clearly the weakest link in welfare state support. Our findings confirm previous research on attitudes towards dimensions of the welfare state when studied separately, although attitudes towards welfare state outcomes were not systematically examined previously.

Third, we found that attitudes towards the dimensions differ between Western/Northern and Eastern/Southern European welfare states. In Western/Northern European countries, respondents are more positive towards the outcomes and efficiency of the welfare state than in Eastern/Southern European countries. In the latter, respondents combine a positive attitude towards the role and goals of government with a more critical attitude towards the welfare state’s efficiency and intended outcomes. In contrast, in Western/Northern welfare states, there is a general welfare attitude that is fundamentally positive or negative. This general welfare attitude partly influences attitudes towards the various dimensions.

How can these differences be explained? Regarding the Eastern European countries, the transition from the communist centrally planned economies towards democratic market economies was a unique historical event. Eastern European welfare states were confronted with a double burden of responsibilities after the transition: protecting people from new and old social risks and coping with the social, political and economic challenges resulting from the transition (Cerami 2007). Because of job losses that accompanied the transition, governments had difficulties ensuring broad coverage of social protection since their protection schemes were based on employee contributions. Rising poverty levels challenged the provision of a reasonable standard of living and new health, pension and unemployment insurance came under financial pressure as a result of rising unemployment. The Eastern European welfare system can therefore not offer the quality and quantity of its Western European counterparts yet (Cerami 2007, 2008). Cerami (2008, p. 18) refers to the dissatisfaction of Eastern European citizens with the quality of their social services. The Southern European welfare states are also known for their limited social protection system, absence of social minimum and small role of the state in providing social services (Arts and Gelissen 2002; Ferrera 1996). In our view, this situation may explain why Eastern/Southern European citizens are more critical towards the performance of their welfare state and, at the same time, endorse more redistribution and more government responsibilities, as a way to close the welfare gap with the Western/Northern European welfare states. In contrast, citizens of Western/Northern European welfare states, who are used to living in a more developed welfare context, believe that more government responsibilities and further redistribution are less necessary.

This study also has some limitations. We were not able to examine attitudes towards the *redistribution* and *welfare mix* dimension. Much research has already investigated these dimensions from a unidimensional perspective, yet it would be valuable to examine them in relation with other dimensions and in a cross-national perspective. In addition, the operationalisation of the *goals* dimension is limited since it does not include attitudes towards the activating role of the welfare state, which is becoming an increasingly important concept. Future cross-national surveys should therefore include (more) measurements related to the various welfare state dimensions and subdimensions that are distinguished in this study. Because of data limitations, we were unable to assess measurement invariance for all dimensions of our model. We therefore need to be cautious in our conclusions regarding country comparisons on these dimensions. Moreover, to make valid cross-national comparisons, we excluded seven countries from our sample because we found evidence against the hypothesis of measurement invariance for these countries. Finally, this study has not investigated explanations for differences in public endorsement for the different dimensions. Future research should investigate which social-demographic and motivational factors explain the variation in the dimensions and how their effects differ between the dimensions.

We conclude with the observation that at least in Europe, we do not face a welfare state legitimacy crisis. The majority of people still support the welfare state and the government’s responsibility to redistribute life chances. In Eastern/Southern European countries, a critical stance towards the level of benefits and services is combined with even larger support for the goals and range of the welfare state. The greatest risk for welfare state legitimacy seems to be perceptions of abuse of welfare benefits, which are rather widely shared among the European people.

## Appendix

See Table 8.

## Footnotes

1 For the exact wording of the 26 survey questions, we referred to the European Social Survey 2008.

2 We concluded that measurement invariance of the latent constructs was violated for four non-European countries (Israel, Russia, Turkey and Ukraine) and three European countries (Ireland, Greece and Romania). We found that their contributions to the Chi-Squared test in the multi-group analyses were substantially higher than for the other countries, which imports misfit due to measurement invariance. Moreover, measurement models of these countries were substantially different from the countries for which measurement invariance holds. In order to be able to make valid cross-national comparisons, we excluded them from our sample.

3 An RMSEA of <0.05 is indicated as a good fit; 0.05 < RMSEA ≤ 0.08 indicates an acceptable fit. CFI must be >0.9 to be acceptable (Byrne 1998). Cheung and Rensvold (2002) have argued that in a multi-group analysis, the CFI, instead of the RMSEA, is the most unbiased fit statistic. Two intercepts are constrained across countries (government responsibility for health care and paid care leave). RMSEA: 0.087, CFI: 0.955.

4 In order to identify the model, correlations between latent factors are added. Two intercepts of outcomes goals, two intercepts of outcomes economic and two intercepts of outcomes moral were constrained across countries, but only one intercept of outcomes policy. Strictly speaking, the dimension ‘outcomes policy’ was therefore not scalar equivalent. RMSEA: 0.056, CFI: 0.916.

5 Factor scores were also estimated and correlated with sum scores: r_*range*_: 0.999, r_outcomes-goals_: 0.988, r_outcomes-policy_ 0.984, r_outcomes-economic_: 0.961, r_outcomes-moral_: 0.972.

6 When we estimate a model that includes the seven countries for which measurement invariance does not hold, the model cannot reach convergence, which presumably indicates a strong misspecification of the model. We interpret this misspecification to be additional evidence that the measurement models for these seven countries are substantially different.

7 Results are available from the authors on request.

8 We notice that the excluded countries, for which measurement invariance did not hold, generally follow this country clustering. Ireland can be placed in the Western/Northern European country cluster; Greece, Romania, Russia, Turkey and Ukraine, in the Eastern/Southern European cluster. Israel fits better in the Western/Northern cluster.

9 The Multi Group CFA of the two groups of countries showed a RMSEA of 0.051 and a CFI of 0.885.
